# Discriminating Healthy Optic Discs and Visible Optic Disc Drusen on Fundus Autofluorescence and Color Fundus Photography Using Deep Learning—A Pilot Study

**DOI:** 10.3390/jcm12051951

**Published:** 2023-03-01

**Authors:** Raphael Diener, Jost Lennart Lauermann, Nicole Eter, Maximilian Treder

**Affiliations:** Department of Ophthalmology, University of Muenster Medical Center, 48149 Muenster, Germany

**Keywords:** deep learning, artificial intelligence, optic disc drusen, visible optic disc drusen, optic disc drusen, deep convolutional neural network, DCNN, inceptionv3

## Abstract

The aim of this study was to use deep learning based on a deep convolutional neural network (DCNN) for automated image classification of healthy optic discs (OD) and visible optic disc drusen (ODD) on fundus autofluorescence (FAF) and color fundus photography (CFP). In this study, a total of 400 FAF and CFP images of patients with ODD and healthy controls were used. A pre-trained multi-layer Deep Convolutional Neural Network (DCNN) was trained and validated independently on FAF and CFP images. Training and validation accuracy and cross-entropy were recorded. Both generated DCNN classifiers were tested with 40 FAF and CFP images (20 ODD and 20 controls). After the repetition of 1000 training cycles, the training accuracy was 100%, the validation accuracy was 92% (CFP) and 96% (FAF), respectively. The cross-entropy was 0.04 (CFP) and 0.15 (FAF). The sensitivity, specificity, and accuracy of the DCNN for classification of FAF images was 100%. For the DCNN used to identify ODD on color fundus photographs, sensitivity was 85%, specificity 100%, and accuracy 92.5%. Differentiation between healthy controls and ODD on CFP and FAF images was possible with high specificity and sensitivity using a deep learning approach.

## 1. Introduction

Optic disc drusen (ODD) are acellular deposits that are located in the optic nerve head of 0.3% to 2.0% of the population [1,2].

In children and younger individuals, ODD are mostly buried deep in the optic nerve head [3,4]. They can be diagnosed using various imaging techniques, such as B-scan ultrasonography or, more recently, swept source (SS) or enhanced depth imaging (EDI) optical coherence tomography (OCT) [5,6]. Most of these cases are asymptomatic [7].

Due to an increase in drusen number, drusen growth or age-related thinning of the overlying retinal nerve fiber layer, ODD become visible with age and can, therefore, be detected on color fundus photography (CFP), fundus autofluorescence (FAF), and ophthalmoscopy [7]. Visible ODD are associated with visual field defects in up to 87% of cases [2,8,9,10]. Consequently, they are associated with high clinical relevance for visual function [11].

Because of the widespread use of multimodal imaging technologies as well as the digital fundus cameras for eye screening programs, there is an increasing amount of data to be analyzed by ophthalmologists, and therefore, a remarkable interest in the automated screening for optic nerve pathologies, such as ODD.

Artificial intelligence using deep learning (DL), a subtype of machine learning (ML), is used to solve complex and large-scale problems, such as speech and image recognition and language processing. The three most popular DL models are recurrent neural networks (RNNs), generative adversarial networks (GANs), and convolution neural networks (CNNs), which are particularly well suited for different tasks depending on their architecture.

RNNs are widely used in natural language processing and speech recognition tasks, where the input data are sequential in nature, such as text or speech. They use feedback connections that allow previous outputs to be used as inputs for subsequent processing, enabling the network to persist information across multiple steps and analyze complex dependencies in the data [12].

GANs have been applied to generative modeling tasks, such as image generation. They consist of two parts, a generator and a discriminator, that compete with each other to generate new data samples that are indistinguishable from real data [12].

CNNs are designed specifically for image classification tasks and are particularly well suited for recognizing patterns and features in images and have revolutionized data processing in medicine, especially in image-centric disciplines [12], such as Dermatology [13], Radiology [14], Pathology [15], and Ophthalmology [12,16]. In this context, CNNs have already been successfully used for automated image analysis using color fundus images for a number of ophthalmologic diseases with high prevalence, including glaucoma [17], diabetic retinopathy [18], and age-related macular degeneration [19].

ML and DL algorithms have several inherent limitations, including the need for very large, accurate datasets for learning. To overcome this limitation, transfer learning, which uses an already pre-trained deep learning algorithm can be used [19,20,21].

The aim of this study was to evaluate the use of a pre-trained CNN for the automated classification of visible ODD and healthy optic discs on fundus autofluorescence (FAF) and color fundus photography (CFP).

## 2. Materials and Methods

This study adhered to the tenets of the Declaration of Helsinki. Informed consent was waived due to the retrospective nature of the study and the fully anonymized usage of the database.

### 2.1. Patient and Image Selection

Patients with a clinical diagnosis of ODD and color fundus photography and fundus autofluorescence image of the optic disc were included in this study. Patients with no evidence of an optic disc pathology as determined by an ophthalmologist were defined as controls.

Images were chosen from a database of the Eye Clinics of Muenster University Hospital, compiled between January 2015 and January 2020. A total of 480 CFP and FAF images of the ODD and control group were used. All images were focused on the optic nerve head and were obtained using the same fundus autofluorescence (Spectralis, Heidelberg Engineering, Heidelberg, Germany) and color fundus photography (Visucam 500, Carl Zeiss Meditec AG, Jena, Germany) device. FAF devices produce greyscale images, whereas CFP devices produce Red-Green-Blue (RGB) images.

Inclusion criteria were selected in which drusen were visible in FAF as hyperfluorescent material. Images with buried optic disc drusen that were only visible in sonography or OCT were excluded.

All images were saved as JPEG files and had an input size of 299 × 299 × 3 pixels.

### 2.2. Deep Learning

Training and validation of the DL model (InceptionV3) were performed using TensorFlow^TM^ (Google Inc., Mountain View, CA, USA), which is an open-source software program developed by Google. It provides a high-level interface for designing and training DL models [20,22,23,24,25]. InceptionV3 is a DCNN designed for image classification tasks that was introduced by Szegedy et al. in 2015 [22]. It uses a modular architecture with multiple parallel convolutional paths and a concatenation layer that merges the result. This allows the network to capture both global and local features in the input image. Each layer takes an input and produces an output, which becomes an input to the next processing layer, creating a deep architecture. In each successive layer, the data were represented in an increasingly more abstract way. All layers, with the exception of the last layer, were pre-trained with an ImageNet [26] data set consisting of more than 14 million images of different objects and scenes. InceptionV3 can be fine-tuned for specific image-classification tasks with smaller datasets, which allows for faster and more accurate results. For this study, the last layer was trained with our ophthalmic dataset [27,28].

**Figure 1:**
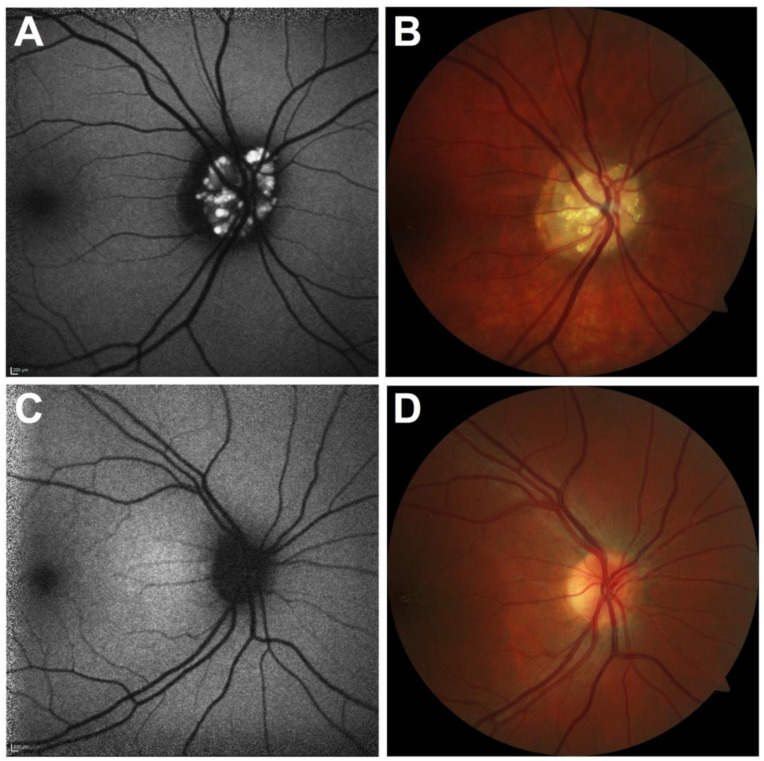
Fundus autofluorescence (**A**,**C**) and color fundus photography images (**B**,**D**) were used independently for training of the two different classifiers.

Two deep learning models were independently trained and validated using 120 FAF photos (ODD: *n* = 60; healthy: *n* = 60) and 120 CFP images (ODD: *n* = 60; healthy: *n* = 60) over the course of 1000 training steps (Figure 1). The training and validation accuracy, as well as the cross-entropy, were calculated in each of the training steps to evaluate the effectiveness of both training strategies. Forty FAF and 40 CFP photos (OOD: *n* = 20, healthy: *n* = 20) were used to assess the performance of both the developed DCNN models once the pre-training was completed (FAF and CFP). The 40 FAF and 40 CFP images used for testing were excluded from the dataset before training and validation of the algorithm were performed. The algorithm, therefore, had no access to the test data set during training and validation. Accordingly, the performance of the algorithm could be tested without bias. 

### 2.3. Statistics

SPSS was used to perform the statistics (IBM SPSS Statistics 23.0; IBM, Armonk, NY, USA). For descriptive statistics, Prism was utilized (Prism 7, GraphPad Software, Inc. San Diego, CA, USA). Data administration was carried out using Microsoft Excel (Microsoft^®^ Excel^®^ for Mac 2011, 14.6.2; Microsoft^®^, Redmond, WA, USA).

Mean differences in the probability scores of the two classifiers were verified with Mann–Whitney U-test for independent samples. The level of significance was defined as *p* < 0.05.

Using a 2 × 2 table, the sensitivity, specificity, and accuracy were computed. Both the DL procedure and the testing were repeated with the same data set to enable the evaluation of the precision of the repeatability of the ODD testing score. Coefficients of variation were computed to evaluate the precision. Bland–Altman plots were employed to evaluate repeatability.

## 3. Results

### 3.1. Performance of the Training Process

**Figure 2:**
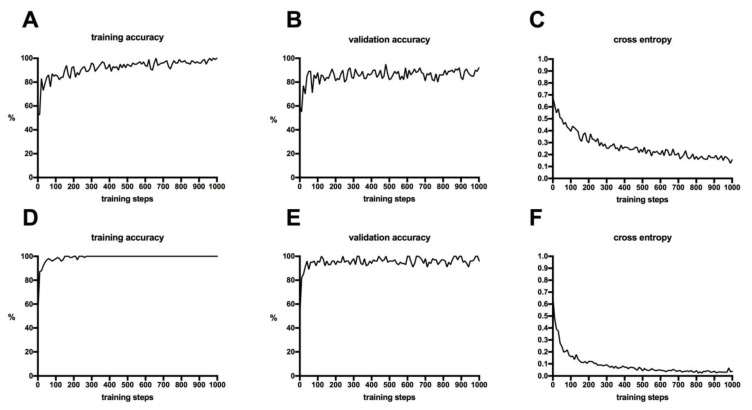
The graphs show the development of the training accuracy, validation accuracy, and cross-entropy of the two classifiers trained with color fundus photography (**A**–**C**) and fundus autofluorescence (**D**–**F**).

Both classifiers for FAF and CFP images had a training accuracy of 100% after 1000 performed training steps. The validation accuracy of the classifier for CFP and FAF images was 92% and 96%, respectively. There were no notable differences in the course of the curves of the training and the validation accuracy. The cross-entropy of both classifiers constantly decreased and was 0.15 (CFP images) and 0.04 (FAF images) after completion of the training process, as seen in Figure 2.

### 3.2. Testing of the Classifiers

All FAF images of both ODD and healthy test patients were correctly diagnosed by the classifier trained on this image modality. Consequently, sensitivity, specificity, and accuracy of this classifier were 100%, as shown in Table 1. The mean ODD testing scores for the ODD testing group’s photos were 0.91 ± 0.15, and 0.05 ± 0.07 for the healthy control group’s images. The mean healthy testing scores for the ODD testing group’s images were 0.09 ± 0.15, and for the healthy control group’s images, they were 0.95 ± 0.07.

All CFP images of the healthy test group were correctly diagnosed by the classifier whose last layer was trained with 120 CFP images. Three CFP images of patients with ODD were misdiagnosed by this classifier. Therefore, this classifier had a sensitivity of 85%, a specificity of 100% and an accuracy of 92.5%, as shown in Table 2.

The mean ODD testing scores were 0.79 ± 0.25 for the images in the ODD testing group and 0.10 ± 0.12 in the healthy control group. The mean healthy testing scores were 0.09 ± 0.15 for the images in the ODD testing group and 0.90 ± 0.12 for the healthy control group.

The difference between the mean testing scores for the differentiation of diseased and healthy optic discs was statistically significant (*p* < 0.001) for both FAF and CFP images.

### 3.3. Repeatability and Precision

The initial computed testing scores and the scores of the repeated testing had a mean coefficient of variation of 0.22 ± 0.59% (FAF) and 3.73 ± 5.83% (CFP), respectively, indicating both classifiers had good precision. Between the two tests, the mean difference had absolute values of 0.001 ± 0.005 (FAF) and 0.006 ± 0.07 (CFP).

**Figure 3:**
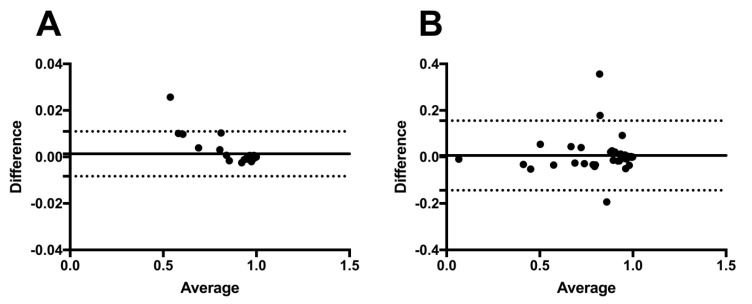
To determine the degree of agreement between the test results from the initial and subsequent deep learning procedures using fundus autofluorescence (**A**) and color fundus photography (**B**), Bland–Altman plots were used. The average difference in ODD score between the two treatments is shown by the solid line. The ranges ([mean of the difference] + 1.96 [standard deviation of the difference]) and ([mean of the difference] − 1.96 [standard deviation of the difference]) are shown by the dashed lines.

The Bland–Altman plots indicate high values of repeatability for both classifiers. The results for the classifier using FAF images were even superior to that using CFP images, as seen in Figure 3.

## 4. Discussion

Machine learning (ML) and deep learning (DL) have increased the possibilities for automatic image analysis in ophthalmology. DL has been successfully used for the automatic detection of diseases with high prevalence, such as diabetic retinopathy [18,29], age-related macular degeneration [27,30], and glaucoma [17], using different image modalities. In this context, it seems plausible to extend the use of DL to other, less frequent diseases, like optic disc drusen (ODD). Our results show that DL is a suitable approach to facilitate image analysis in this rare diagnosis.

Many of the DL studies mentioned above achieved a sensitivity and specificity of more than 90%, but in most of them, thousands of images were necessary to train the algorithms [17,18,27]. Despite the small amount of data used due to the low prevalence of ODD, especially when compared to widespread diseases, the classifiers used in this study achieved an accuracy of 100% and 92.5%, respectively. Additionally, this approach has already been successfully applied in pre-published work [27,28,31].

Shah et al. were able to show in a preliminary study that DL can be effectively used with a small amount of data for training to classify normal OCT scans and those from patients with Stargardt’s disease at different stages and, therefore, characteristic of the disease [32]. Training and testing data were composed of 749 OCT B-scans of only 93 individuals. Similar to our study, a CNN architecture pre-trained with the ImageNet dataset was used and achieved sensitivity and specificity levels of over 95% [26].

In our study, an even smaller amount of FAF and CFP images was used, achieving similar results with a sensitivity of 100% for both classifiers and a specificity of 100% with fundus autofluorescence and 85% with color fundus imaging.

Different aspects could explain why a similar performance of the algorithm was achieved in this study although an even smaller data set was used.

First, the use of multiple images of a single eye potentially reduced the diversity within the data set of Shah et al. [32]. In our study, only one image of a single eye was used. Second, the use of data from one disease at various stages of Stargardt’s disease leads to a limited ability of the classification model to differentiate images with a milder disease phenotype. In contrast, our study only considered images with superficial drusen. This makes it easier for the algorithm to learn specific aspects of this disease subgroup, although its field of application is limited to a smaller patient collective.

In our study, we used FAF and CFP images to analyze ODD because first, superficial ODD visible in FAF have a higher risk of causing a visual field defect compared to buried ODD [11], and second, CFP imaging is a widely used image modality in screening examinations. Thus, the algorithm could be used as s screening tool for visible ODD on color fundus photographs to then initiate further diagnostics, such as performing a visual field examination.

**Figure 4:**
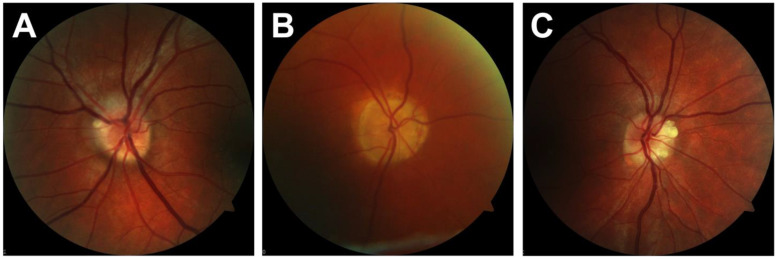
Three ODD images were incorrectly identified as healthy optic discs. This might be due to (**A**) low contrast according to the Juvenile reflexes and mostly buried optic discs drusen, (**B**) low contrast due to low image quality, (**C**) unclear.

Comparing the results of FAF and CFP image analysis, patterns of ODD seemed to be easier to recognize on FAF images for the algorithm. This can be seen in the relatively flatter training accuracy curve in Figure 2 and is an indicator of a higher learning rate. Additionally, the DCNN is able to distinguish more clearly between healthy subjects and ODD on FAF images. All ODD eyes were correctly identified on FAF images, whereas three CFP images were misdiagnosed as being healthy (Figure 4). This may indicate that FAF is superior to CFP in the identification of superficial optic disc drusen. This seems plausible since visible drusen in FAF are clearly distinguishable by autofluorescence [7].

However, RGB images (CFP) are 3-channel color images, while greyscale images (FAF) have only one channel that represents the intensity of the image. When using InceptionV3, the model would expect an input image with the same number of channels as its pre-trained weights. If a grayscale image is fed as an input, it would have to be first converted to an RGP image by repeating the single channel across the three channels. Thus, it could be expected that the DCNN might perform better when given RGB images as input compared to grayscale images. However, if the FAF images contain sufficient information for the task, they may even outperform RGB images, which is the case in our study [33].

Three CFB images were misdiagnosed by the classifier (Figure 4). Due to the black box formation of DCNN the reasons for misdiagnosis of the images by the classifier can only be suspected. However, one reason for this could be that in these three cases, the drusen are not clearly delineated on fundus photographs despite their visibility in fundus autofluorescence.

Even though the applicability for FAF images was better, the CFP images analysis also showed promising results. Automated analysis of CFP images will probably play an even more important role in everyday clinical routine. In contrast to FAF, CFP imaging is a widespread procedure in screening, even without symptoms, in many in- and outpatient settings. The increasing usefulness of fundus imaging offers a vast amount of data that clinicians must thoroughly assess quickly. Similar to computer-assisted detection systems created to help radiologists interpret medical pictures, DL methods, as applied in this study, could help radiologists with the diagnosis and treatment of optic disc illnesses [14]. This could increase the usefulness of screening examinations in general and help to ensure that the data collected are actually fully evaluated and a true benefit for the patient can be derived.

In a recent study, Milea et al. used a deep learning system to detect papilledema on color fundus photographs using a dataset of 14,341 images. They reached sensitivity levels of 96.4% and specificity of 84.7% [34]. Here, ODD were analyzed as a part of a group of “Disks with Other Abnormalities” and were, therefore, not discussed separately. However, the performance results of the algorithms are comparable [34].

This study was limited by different aspects. First, by training the DCNNs exclusively with visible ODD, the algorithms presented here have questionable relevance to everyday clinical practice. For an ophthalmologist, detecting visible ODD, especially using FAF images, is, in most cases, very simple. Therefore, the high specificity and sensitivity values achieved here are not surprising. In contrast, the detection of buried optic disc drusen and its differentiation from other optic disc pathologies, such as optic disc edema, is both highly clinically significant and challenging. In order to support ophthalmologists in their decision-making based on artificial intelligence in everyday clinical practice, further studies are necessary, including buried optic disc drusen. In this pilot study, however, the primary aim was to detect superficial drusen. The classification of deep ODD and its differentiation from other optic nerve pathologies is planned in a follow-up study.

Second, each of our DL classifiers was trained and tested on FAF and CFP images from a single device type. Therefore, the applicability to FAF and CFP images from other devices is unknown. However, we believe that image data from different devices can be used after prior alignment to uniform recording conditions.

Third, the image data set for this study was small compared to other AI studies in the field of ophthalmology. However, as explained above, this can also be seen as a strength of our approach since it can be difficult and time-consuming to build up large data pools, especially for rare diseases. Therefore, algorithms that make reliable statements based on smaller data sets offer an exciting perspective. Maybe, the results of our testing will even improve with a higher amount of data.

Finally, overfitting is a risk associated with using a small dataset to train a DCNN. This can happen if the model is trained with only a few images or with a large number of training steps. The risk is that the model corresponds too closely to the training data and fails to make reliable predictions on new data. In other words, the model is learning patterns that are unique to the training data but irrelevant to other data. The capacity of the DCNN to detect unseen images decreases with subsequent training steps after an initial improvement. Based on the training and validation accuracy curves, an increasing gap is formed between the training and validation accuracy curves. There were no significant differences in the course of the curves of training and validation accuracy in this study, indicating that neither model is overfitting.

## 5. Conclusions

In conclusion, we were able to demonstrate that it is possible to use DL classification models to differentiate between normal FAF and CFP images and those from patients with superficial ODD using a transfer-learning-based DL algorithm.

FAF images seem to be superior to CFP images in the diagnostics using our DL approach. However, the analysis of CFP images also showed promising results. Prospective studies will be crucial for clinical translation and will hopefully confirm and improve our results.

We hypothesize that the general principle demonstrated in this study can be applied to other optic disc abnormalities with a lower prevalence.

## Figures and Tables

**Table 1:** All fundus autofluorescence images of patients with ODD and normal optic discs were correctly identified, therefore, the sensitivity and specificity of the classifier were 100%.

	ODD Testing Group	Healthy Testing Group
Positive	*n* = 20	*n* = 0
Negative	*n* = 0	*n* = 20

ODD = Optic Disc Drusen.

**Table 2:** All color fundus photography images from healthy patients were correctly identified, whereas 3 CFP images from patients with ODD were misdiagnosed. Therefore, the sensitivity was 85%, and specificity was 100%.

	ODD Testing Group	Healthy Testing Group
Positive	*n* = 17	*n* = 0
Negative	*n* = 3	*n* = 20

ODD = Optic Disc Drusen.

## Data Availability

Not applicable.
